# Medical service demand forecasting using a hybrid model based on ARIMA and self-adaptive filtering method

**DOI:** 10.1186/s12911-020-01256-1

**Published:** 2020-09-19

**Authors:** Yihuai Huang, Chao Xu, Mengzhong Ji, Wei Xiang, Da He

**Affiliations:** 1 Faculty of Mechanical Engineering and Mechanics, Ningbo University, Ningbo, 315211 China; 2 Institute of advanced energy storage technology and equipment, Ningbo University, Ningbo, 315211 China; 3 Yinzhou District Maternal and Child Health Care Hospital, Ningbo, 315211 China

**Keywords:** Time series, ARIMA model, Self-adaptive filtering, Hybrid forecasting model, Medical forecasting

## Abstract

**Background:**

Accurate forecasting of medical service demand is beneficial for the reasonable healthcare resource planning and allocation. The daily outpatient volume is characterized by randomness, periodicity and trend, and the time series methods, like ARIMA are often used for short-term outpatient visits forecasting. Therefore, to further enlarge the prediction horizon and improve the prediction accuracy, a hybrid prediction model integrating ARIMA and self-adaptive filtering method is proposed.

**Methods:**

The ARIMA model is first used to identify the features like cyclicity and trend of the time series data and to estimate the model parameters. The parameters are then adjusted by the steepest descent algorithm in the adaptive filtering method to reduce the prediction error. The hybrid model is validated and compared with traditional ARIMA by several test sets from the Time Series Data Library (TSDL), a weekly emergency department (ED) visit case from literature study, and the real cases of prenatal examinations and B-ultrasounds in a maternal and child health care center (MCHCC) in Ningbo.

**Results:**

For TSDL cases the prediction accuracy of the hybrid prediction is improved by 80–99% compared with the ARIMA model. For the weekly ED visit case, the forecasting results of the hybrid model are better than those of both traditional ARIMA and ANN model, and similar to the ANN combined data decomposition model mentioned in the literature. For the actual data of MCHCC in Ningbo, the MAPE predicted by the ARIMA model in the two departments was 18.53 and 27.69%, respectively, and the hybrid models were 2.79 and 1.25%, respectively.

**Conclusions:**

The hybrid prediction model outperforms the traditional ARIMA model in both accurate predicting result with smaller average relative error and the applicability for short-term and medium-term prediction.

## Background

Public healthcare agencies always find themselves in challenging timely and qualified delivery of medical services. The medical resources allocation is increasingly concerned by the management of healthcare service provider since it is directly related to the timely delivery of medical services. The reasonable allocation of medical resources is a scientific decision considering the changes in medical service needs arising in regional population. Good understanding on the medical service demand not only calls for analysis on the current and historical amount of the medical treatment delivered, but also relies on accurate predicting of the trend in the near future. Such trends provide invaluable information for needs assessment, resource planning, facilities evaluation and policy formulations. Therefore, a reliable health demand forecasting (e.g. the outpatient visits in different departments of a hospital) can create alerts for the management of patients’ overflows and scientifically allocate critical medical resources so as to reduce the costs in supplies and staff redundancy.

At present, compared with hospitals in western countries, most of large general hospitals and specialist hospitals in China are still operated in a kind of “walk-in” outpatient instead of “booking” outpatient. Such “walk-in” outpatient leads to a serious “overcrowd phenomenon” which is the main contribution factor to the patients complain on public healthcare service. Both the increasing number of outpatient visits and crowding make a negative impact on quality of medical service. To solve such problem, in long term, China strongly encourages patients to receive normal medical service in different levels of public healthcare institutions like community health center, sub-district/district hospital and to book for special medical treatment in large general hospital by referring. The other more direct (short-term) solution is to configure the healthcare service provider’s resource matching its real demand. Using maternity and child healthcare service as example, if medical service demand can be clearly known in advance, some routine checks can be assigned to district-level maternal and child health care center (MCHCC) instead of going to large hospitals for examination. Then the resources in district-level MCHCCs can be fully utilized and at the same time the overcrowded phenomenon in large hospital can be alienated as well.

The current challenge is to forecast the individual medical service demand trend based on the time series historical data so that different levels public healthcare institutions could arrange resources and prepare in advance. The accurate healthcare forecasting is important to improve the management level of medical institutions especially for those hospitals with mainly “walk-in” outpatient.

Healthcare forecasting is about predicting future health services, healthcare needs and rates of utilization of services based on a foreknowledge acquired through a systematic process. In this work, it is focused on the medical service demand forecasting. In the field of medical service demand prediction, several previous studies were published in the prediction of daily emergency department (ED) visits [1–5], and the incidence of infectious diseases. Most published literatures using time series were developed for short-term forecasting. The time series analysis is the best tool for forecast the trend and is find out a pattern in the historical data and then extrapolate the pattern into the future. The predicting accuracy for available time series models is normally acceptable, when there exist wide variation and large fluctuation or extreme data, the predicting error is not satisfied. The purpose of this paper is to propose a hybrid forecasting method which integrates two traditional approaches to obtain a more reliable forecast for medical service demand forecasting. The hybrid forecasting model is applied to a district-level MCHCC to predict the daily outpatient visits in the two main departments of prenatal examination and B-ultrasound examination. The remainder of this paper is organized as follows: The related literature researches are summarized in section 2. Traditional ARIMA, self-adaptive method and the hybrid forcasting model are described in detail in section 3. The verification of the hybrid model by several test cases from both time series data library and literature works are implemented in section 4. Section 5 explores the application in forecasting the outpatients visit in the prenatal and B-ultrasound examinations. Finally section 6 summarizes the main conclusions and future prospects of this article.

## Literature

For the past few years, there has been increasing attention on the time series models to predict medical services demand. Among those studies, two categories of the prediction methods are commonly used, the statistic-based and the AI-based. The former statistic-based models include auto-regressive integrated moving average (ARIMA) models [5–11], moving average (MA) method and exponential smoothing (ES) method [3, 12, 13]. The latter AI-based models include artificial neural networks (ANN) [14–17] and support vector machines (SVM) [18].

In the study of statistic-based prediction methods, time series models are widely used since time series patterns [4, 5] can better capture short-term fluctuations [6, 19]. ARIMA has advantage in systematic investigation of time series data to obtain meaningful statistical and mathematical interpretations of the series. Hence it is the most popular and direct prediction methods using time series data in healthcare field. The main ARIMA forecasting application involves outpatient visits [4, 10, 11, 20], ED visits [3, 5, 9, 12, 13, 21, 22], hospital discharge [2], etc. Schweigler et al. [9] studied the short-term prediction accuracy of the emergency room hospitalization rate using ARIMA and the traditional historical average model. Han et al. [10] used the ARIMA model to predict the monthly outpatient visits of a hospital in Sichuan. Some researchers also used the ARIMA model to forecast the incidence of infectious diseases [7, 8, 23–25]. Wang et al. [7] used ARIMA model to predict the incidence of hepatitis B; Peng et al. [24] established a seasonal ARIMA model based on historical data of in Jiangsu Province to control the development trend of the epidemic. Han et al. [25] developed and validated a predictive model for outbreaks of respiratory infectious diseases through the ARIMA model, and obtained a comprehensive monitoring and prediction model based on the number of emergency visits.

Recent efforts of ARIMA in time series forecasting research are focused on the more accurate approach improving the traditional ARIMA or integrating with several other models or data preprocess technique. Kadri et al. [26] proposed a statistical method based on the vector autoregressive moving average (VARMA) model and a multivariate time series prediction model for short-term prediction of daily attendance of ED. Lim et al. [27] used the ARIMA model with multiple linear regression model with ARIMA errors, with or without the inclusion of influenza predictors to predict the number of emergency department (ED) admissions in Singapore due to pneumonia. The data results show that the MAPE of the two multiple linear regression models with ARIMA error are less than 10%. Luo et al. [11] proposed a new prediction model based on a seasonal ARIMA model and a single exponential smoothing model, considering the periodicity and the effect of the day of the week on the daily outpatient diagnosis of the hospital. The model has more better predict performance than a single model. Aghelpour et al. [28] compared the SARIMA model with the SVR and its combined model. The SARIMA model is currently used in multiple forecasting fields and is a time series that can describe unstable behavior in different seasons.

Since some complex time series contain nonlinear components, many researchers introduced artificial intelligence to time series medical prediction, e.g. artificial neural networks (ANN) [14–17], support vector machines (SVM) [18, 29], etc. Gul and Guneri [14] predicted patient length of stay (LOS) based on patient age, sex, mode of arrival, treatment unit, medical tests and inspection in the ED using ANN. Yousefi and Ferreira [15] provided a ANN-based forecasting tool in order to predict the number of visitors in an emergency department in Hospital Risoleta Tolentino Neves. Both feed forward neural network (FWNN) and recurrent neural network (RNN) presented reasonable predictions of the ED visits in a one-week-ahead time horizon, and RNN slightly outperform the FWNN for this task. Diao et al. [18] established SVM to predict the incidence rate by extracting the characteristics of viral hepatitis data and reported that the time series data of viral hepatitis incidence rate is more complicated, and the fitting effect of SVM model is better than ARIMA model. Furthermore, several researches mentioned that due to the complexity of some time series data consisting of linear and nonlinear modes, it may be difficult to obtain higher prediction accuracy using only linear models or neural network models. The hybrid model or the integration with data decomposition emerged in time series forecasting problem recently. Purwanto et al. [30] present a dual hybrid forecasting model based on a combination of linear regression, neural network and fuzzy models to finally yield a qualitative output for decision making in healthcare management. Khaldi et al. [31] studied the effect of multi-step prediction strategies on the performance of long- and short-term recursive neural network models (SRN, LSTM, and GRU), and finally proposed corresponding strategies for the three models. Bento et al. [32] proposed a novel Bat-inspired hybrid method integrating bat algorithm and scaled conjugate gradient algorithm to improve the learning ability of neural networks. Khaldi et al. [16] studied the artificial neural network (ANN) combined with a signal decomposition technique to predict the weekly emergency department arrivals in hospitals. The time series is decomposed into several sub-signals, and each sub-signal is modeled using a different ANN model. They proved that data decomposition is a powerful tool for data preprocessing, which can improve the generalization ability of ANN while reducing the problem of overfitting. Huang et al. [17] used a hybrid method of empirical mode decomposition and back-propagation artificial neural network optimized with particle swarm optimization to predict outpatients.

As we known, a prediction model can be qualified as good model not only because of its high prediction accuracy but also on good understanding of the data features. Moreover, the model should also be implemented easily even for complicated time series. In this work, we attempt to simplify the outpatient visits prediction problem and improve the forecasting accuracy by capturing the intrinsic fluctuating characteristics of the hospital’s daily outpatient visits.

Hospital’s daily outpatient visit forecasting is a typical time series prediction problem. The randomness and periodic fluctuation characteristics have the greatest impact on the prediction accuracy. Since the ARIMA model has advantages in its simplicity and can directly use only endogenous time series data as input, it is widely used in time series forecasting. This article focuses on outpatient visit forecasting problem in hospitals with mostly “walk-in” outpatient and aims to develop an efficient method that can predict trends in the number of daily visits. In order to achieve more accurate prediction results, we first apply the ARIMA model to identify the periodicity and autocorrelation in time series data. The prediction of time series by a traditional ARIMA model is only applicable to short-term predictions (in the next few days), and the prediction accuracy is low for medium- and long-term predictions (more than one month). The adaptive filtering method is further introduced to adjust the parameters in the ARIMA model so as to compensate for the shortcomings of low accuracy for long-term prediction in the traditional ARIMA model. Finally, test cases in benchmark library and literature are used to validate the proposed hybrid model. The hybrid model is applied to predict the outpatients visit in the prenatal and B-ultrasound examinations from January 2017 to March 2018 in Ningbo Yinzhou District Maternal and Child Health Care Hospital.

## Methods

The basics of the traditional ARIMA and the adaptive filtering is presented and is discussed each advantage and disadvantage, and a hybrid model integrating both method is proposed with detail flow and verification in this section.

### ARIMA model

ARIMA model is generally denoted ARIMA (p, d, q) and is a systematic approach for predictive modeling of both stationary time series and non-stationary time-series data. For non-stationary time series data, its mean and variance are unstable, and it is generally converted to the stationary time series first by differential operation, and then the stationary time series data is used to establish the ARMA model. The general expression of ARIMA model is:

1 $$ {x}_t={\phi}_0+{\phi}_1{x}_{t-1}+\dots +{\phi}_p{x}_{t-p}+{\varepsilon}_t-{\theta}_1{\varepsilon}_1-\dots -{\varepsilon}_q{\varepsilon}_{t-q} $$

The value of time series at time t is a multiple linear function of the historical data of the previous p period (*x*_*t* − 1_, *x*_*t* − 2_, …, *x*_*t* − *p*_) and the prediction error of the previous q period (*ε*_*t* − 1_, *ε*_*t* − 2_, …, *ε*_*t* − *q*_). The error term *ε*_*t*_ are generally assumed to be independent, identically distributed variables sampled from a normal distribution with zero mean.

The modeling process is divided into four steps:

Step 1: Stationarizing the Time Series: The method of stationarizing is to difference time series data, where d is the degree of differencing. Data preprocessing is required for time series, that is, stationary and randomness tests. There are two methods for checking the stationary of time series. One is the observation method, which is based on the characteristics of the time series graph and the autocorrelation graph. The other is the method of constructing statistics test, so called unit root test. Pure randomness test, also known as white noise test, is usually tested Q statistics and LB statistics.

Step 2: ARIMA model identification: It is to determine the order of model, that is to determine the order of the auto-regressive part (p), the order of difference (d), and the order of the moving average part (q). The d is already determined in step 1, while p and q are determined according to nature of the auto-correlation function (ACF) and partial auto-correlation function (PACF). In case of several (p,q) existing, AIC (Akaike Information Criterion) or BIC (Schwartz Bayesian Criterion) for different (p, q) are calculated by eq. (2) (3) and the (p, q) with smallest AIC or BIC value is determined to be the final order parameter.

2 $$ {\overline{x}}_{t+1} $$

3 $$ t+1 $$

Where n is the sample size, σ^2^ is the sum of the squared residuals, {p, d, q} are model parameters.

Step 3: Estimation of coefficients and validation: Estimation of coefficients is performed using the least square method or the maximal fitness method. Generally, the rationality of the model is to test the standard model fitting residuals. If the fitting residuals of the model satisfy the normal distribution with zero as the mean and the autocorrelation coefficient for any lag order residuals is zero, the model is regarded as the optimal model for time series. If the model test is unreasonable, return to step 2 to re-identify the model.

Step 4: Application of the model: The final ARIMA model is ready to make prediction on future time points by rolling one-step forecasting.

### Self-adaptive filtering method

The Self-adaptive filtering method is based on a weighted average of historical time series observations. If the weights are all equal, the adaptive filtering method is the moving average method. According to the mathematical optimization principle, the weights in the moving average model are adjusted to reduce the prediction error [33]. This method is more common in economic fields and engineering testing applications [34], such as stock trend, futures market forecast [35], but rarely used in the healthcare field. Its general expression is:

4 $$ {\overline{x}}_{t+1}={w}_1{x}_t+{w}_2{x}_{t-1}+\dots +{w}_N{x}_{t-N+1}=\sum \limits_{i=1}^N{w}_i{x}_{t-i+1} $$

Where ${\overline{x}}_{t+1}$ is the predicted value of the *t* + 1 period of the original data of the time series, *w*_*i*_ is the weight of the *t* − *i* + 1 period data, *x*_*t* − *i* + 1_ is the observation value of the *t* − *i* + 1 period of the time series data, and *N* is the number of valuable previous period of the time series for prediction. The core idea is the determination of its weights by adaptive error tracking and immediate compensating. The weights are kept adjusting according to the prediction error feedback in each iteration of all training data until a set of “best” weights is found when the error is converged.

The detail process is as follows:

Step1: Determine N and the initial weights.

Usually N is set as the cycle length if the time series has cyclic feature, or is determined by autocorrelation analysis if no obvious cycle existing. A set of initial weights *w*_*i*_(*i* = 1, 2, …, *N*) is set equally as 1/N, that is, the basic moving average;

Step 2: Predicting and error tracking.

The predicted value ${\overline{x}}_{t+1}$ is calculated according to the formula (4) and the error is calculated between the actual value and the predicted value:

5 $$ {e}_{t+1}={x}_{t+1}-{\overline{x}}_{t+1} $$

Step 3: Adjusting weights in iteration.

The weight is adjusted iteratively according to the error *e*_*i* + 1_, the observation value and a learning constant *k* in order to compensate the prediction error. The iterative formula of weight is:

6 $$ {w}_i^{\hbox{'}}={w}_i+2{ke}_{t+1}{x}_{t-i+1} $$

The formula comes from the steepest descent method approximation. According to the principle of optimization, it takes the minimum standard deviation of prediction as the objective function. According to the literature [8], the sufficient condition for the convergence of (6) is:

7 $$ k\le \frac{1}{\underset{n}{\max}\left\{\sum \limits_{i=1}^n{x}_i^2\right\}} $$

The denominator represents the mean square sum of n observations in historical time series data. A good k value not only reduces the number of iterations, but also ensures the error is minimized.

The weights are adjusted until a set of “best” weights are found to minimize the error. Assume total training time series data is M, such weights adjusting repeat (M-N) times in one round of iteration. Then the last set of weights obtained in this iteration is set as the initial weight of the next iteration. The iteration round is stopped until the error is converged, and then the set of “best” weight is obtained.

Step 4: Predicting by using the best weight.

Such “best” weights will be used for forecasting according to the formula (4).

There are two advantages of adaptive filtering. First, it is simple and the number of “weights” and the learning constant k can be selected according to the needs of the researcher to control forecasting. Secondly, the method uses the observation of all series to find the “best” weight, and keep updating the weight with the change of historical data, making the prediction more accurate. Moreover, the “weight” in the adaptive filtering method is arbitrary and without any constraints, that is, the sum of the adjusted weights is not necessary to be equal to 1 and even can be negative.

### ARIMA-self-adaptive filtering hybrid forecasting model

Traditional ARIMA modeling normally has good accuracy only for short-term prediction but the prediction error increases with the increasing of prediction horizon. However, the adaptive filtering is just perfect to reduce prediction errors by iteratively adjusting the “weight”. Hence, the integration of these two methods can make the short-term prediction even more accurate than the traditional ARIMA model and simultaneously keep the good prediction accuracy even when the prediction horizon increases.

**Fig. 1:**
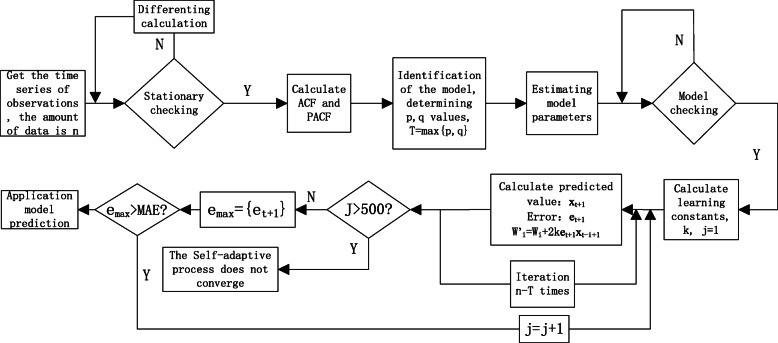
The flow chat of hybrid forecasting model Based on ARIMA and Self-adaptive Filtering Method. ACF: Auto-correlation function, PACF: Partial auto-correlation function, MAE: Average absolute error

One of the steps in the traditional ARIMA modeling process is the estimation of model parameters, i.e. *φ*_1_, *φ*_2_, …*φ*_*p*_, *θ*_1_, *θ*_2_, …, *θ*_*q*_ in eq. (1). Since eq. (1) can be viewed as the weighted polynomial, *φ*_1_, *φ*_2_, …*φ*_*p*_, *θ*_1_, *θ*_2_, …, *θ*_*q*_ turn to be the “weights” in the adaptive filtering method. After the third step of ARIMA modeling, we use the estimated parameter *φ*_1_, *φ*_2_, …*φ*_*p*_, *θ*_1_, *θ*_2_, …, *θ*_*q*_ as the initial weight and use self-adaptive filtering idea to adjust the weight parameters so that the prediction error is reduced as much as possible. Finally, a set of “best” parameters is fitted back to the ARIMA model for prediction. The model is implemented as shown in Fig. 1 and the convergence is measured by the minimum absolute error (MAE) of the ARIMA prediction results. Figure 1 gives the flow chart of this hybrid forecasting method. The specific modeling steps are as follows:

Step 1: First, the traditional ARIMA model is determined based on the obtained time series observations, involving stationary checking, ACF and PACF calculating, and parameters estimation, and obtain the ARIMA(p,d,q) and the initial estimated parameters *φ*_1_, *φ*_2_, …*φ*_*p*_, *θ*_1_, *θ*_2_, …, *θ*_*q*_.

Step 2: Use the ARIMA model for prediction, and calculate the absolute error *e*_*t*_ of the predicted value, and determine the MAE of all predicted values in one iteration round for checking the error convergence.

8 $$ {e}_t={x}_t-{\overset{\frown }{x}}_t $$

Where: *e*_*t*_ is the prediction error at time t, *x*_*t*_ is the actual observed value at time t, ${\overset{\frown }{x}}_t$ is the predicted value.

Step 3: Start adaptive filtering to keep adjusting the parameters and iteratively calculate the “best” parameters. The condition for stepping out of the iteration round loop is either the error is converged or enough iteration rounds have finished.

Step 4: Return the “best” parameters to the ARIMA model for model prediction.

## Results

### Verification of the hybrid model

In order to validate the proposed hybrid forecasting method, test cases from benchmark library and literature are used. We selected different time series data from The Time Series Data Library (TSDL) (https://datamarket.com/data/list/?q=provider:tsdl) created by Monash University in Australia for model verification. The selected test cases cover time series data of stationary, with uptrend or downtrend, with both periodic and trend, etc. and the prediction results of the hybrid model are compared with the traditional ARIMA model to evaluate prediction accuracy and applicable range. Meanwhile, we referred weekly ED visits data in Khaldi et al. [16], and applied hybrid forecasting model for prediction. We compare the prediction results with the ARIMA, ANN, and ANN with data decomposition in the paper.

#### TSDL case verification

**Fig. 2:**
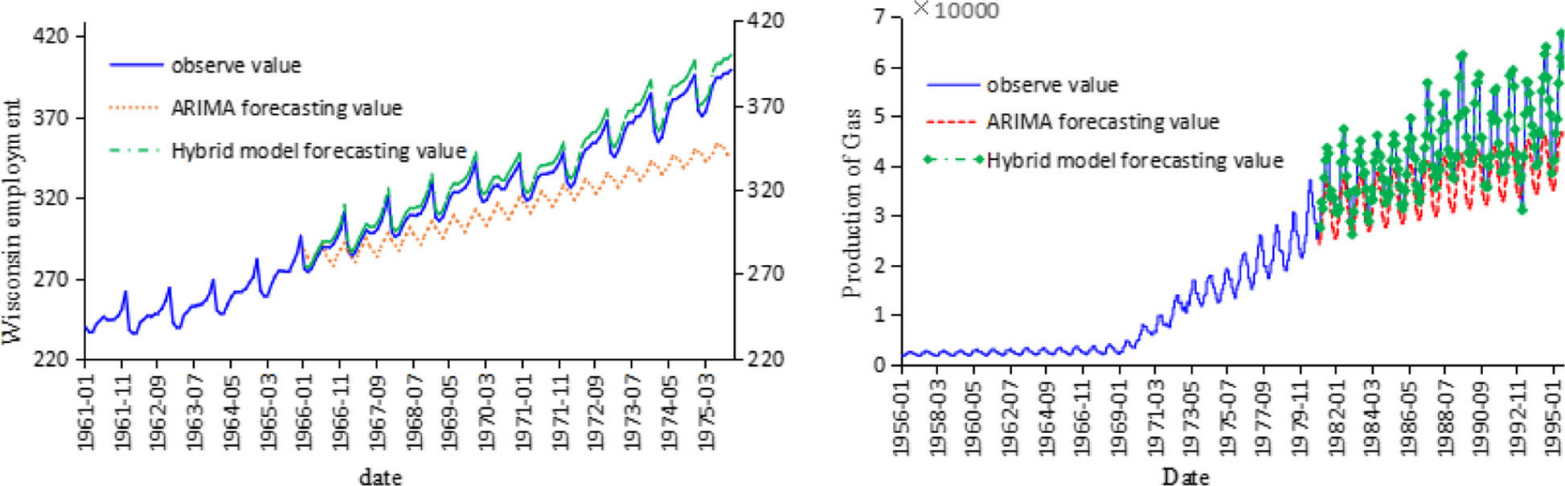
Time series of observed and predicted monthly average employment from 1962 to 1975 and values of monthly average gasoline production from 1956 to 1995. ARIMA: auto-regressive integrated moving average, Hybrid model: ARIMA-self-adaptive filtering hybrid forecasting model

A total of 13 sets of time series data of different sizes (Table 1) selected in TSDL are classified into four categories: Stationary time series; with both periodicity and trend; rising trend; the downward trend. For the time series with both periodic and trend features, two types are further selected. One is that the amplitude of data fluctuations in a period is constant over time (e. g Wisconsin employment time series shown in Fig. 2). The other is that the fluctuations of the data vary over time (e. g, Monthly production of Gas in Australia shown in Fig. 2). For time series with only rising or falling trend, we also further choose two types of data, one is without fluctuation in the rising or falling trend of the data or the fluctuation amplitude is negligible. The other type is with fluctuations in the data upward or downward trend.

**Table 1:** Comparison of ARIMA model and hybrid model predictive value PE

type of data	CASE (The amount of data, the amount of observation set) & ARIMA(p,d,q)	ARIMA forecasting				ARIMA and adaptive combination forecasting				Number of iterations	Iteration time (s)
		PE_max_ (%)	PE_min_(%)	MAPE(%)	σ _PE_(%)	PE_max_ (%)	PE_min_ (%)	MAPE(%)	σ _PE_(%)		
Stationarity	Case 1 (348,336) ARIMA(3, 0, 3)	106.73	0.34	38.4	29.2	0.67	0.00	0.101	0.199	666	2.28
	Case 2 (384,372) ARIMA(2, 0, 5)	134.71	3.25	40.91	35.6	0.62	0.07	0.14	0.19	2208	3
	Case 3 (852,840) ARIMA(7, 0, 7)	49.9	1.8	15.2	13.9	0.192	0.003	0.05	0.06	1670	5
Cyclical and trending	Case 4 (168,156) ARIMA(5, 1, 5)	11.0	0.1	3.1	2.9	0.08	0.01	0.02	0.02	13,892	7
	Case 5 (476,464) ARIMA(6, 1, 4)	8.25	0.36	4.67	0.23	1.29	0.002	0.42	0.47	30,492	10
	Case 6 (252,240) ARIMA(5, 1, 5)	19.5	1.33	9.1	6.01	2.4	0.0003	0.41	0.74	1410	5
	Case 7 (178,168) ARIMA(5, 1, 5)	3.63	0.33	1.8	1.15	1.266	0.0002	0.509	0.523	14,507	13
Upward trend	Case 8 (82,77) ARIMA(4, 1, 4)	13.59	2.34	7.33	3.88	0.005	0.004	0.005	0.001	803	3.9
	Case 9 (418,406) ARIMA(3, 1, 6)	3.58	0.17	1.2	0.91	0.103	0.003	0.052	0.029	8932	6.8
	Case 10 (43,38) ARIMA(1, 1, 3)	10.62	6.95	8.5	1.24	8.13	0.07	1.8	3.18	2805	4
Downward trend	Case 11 (304,296) ARIMA(6, 1, 6)	16.81	2.94	9.78	5.29	1.204	0.002	0.39	0.39	13,050	10
	Case 12 (102,92) ARIMA(1, 0, 0)	4.83	0.36	2.6	1.62	3.62	0.21	1.7	0.99	6622	3.054
	Case 13 (65,55) ARIMA(3, 1, 3)	6.89	1.08	4.46	1.6	5.9	0.64	3.89	1.77	4017	3

*ARIMA* auto-regressive integrated moving average, *PE*_*max*_ Maximum percentage error, *PE*_*min*_ Minimum percentage error, *MAPE* mean absolute percentage error, *σ*_*PE*_ value of the standard deviation of the PE

The time series historical data of each case is divided into two parts, the observation set and the verification set. The observation set is used to predict the latter data and compare the predicted value with the true value of the verification set. The traditional ARIMA model and the hybrid model are used to predict the 13 sets of data, and the relative prediction errors (PE) of both models are calculated. Several measurements including the maximum, minimum and mean absolute percentage error (MAPE) in the PE, and the value of the standard deviation of the PE are summarized in Table 1. The basic ARIMA model (p,d,q), the additional self-adaptive iteration and its computation time are also given in Table 1.

The comparison results of the 13 sets of time series cases from TSDL are summarized in Table 1. It can be seen that in the short-term prediction, the PE obtained by the ARIMA model in the stationary time series is larger. The MAPE of the ARIMA model for predicting stationary time series is generally between 10 and 30%. For the non-stationary time series, the prediction accuracy of the ARIMA model is higher, and the predicted MAPE is between 1 and 5%. The proposed hybrid prediction model has a much better prediction accuracy of over 97% or even approaching to actual value (such as examples 3, 4, and 8) for forecasting time series with periodicity and trend,. As to the standard deviation of relative error, we find that when the amount of time series sample data is small (such as case 10 and case 13), the *σ*_*PE*_ value of the hybrid prediction model is larger than that of the ARIMA model. For the other cases (with larger data volumes), the hybrid prediction model all gives a smaller *σ*_*PE*_ than the ARIMA model.

The additional computation time mainly depends on the iterations in searching optimized parameters. Therefore, computation efficiency is different case by case in those 13 test cases. The iterations and the additional computation time are provided in the Table 1. The additional computations in those 13 cases range from few seconds to 13 s, depending on the number of convergence iterations in finding the optimal parameters.

#### Literature test case comparison

In this section, the hybrid model was applied to a literature test case by Khaldi et al. [16] for further verification and comparison. The time series data of weekly emergency department visits in the university hospital Hassan II of Fez city of Morocco from January 2010 to December 2016 were used as input to the forecasting models. The hybrid model is compared with the traditional ARIMA, ANN (Feedforward neural network), ANN combined with data decomposition technique called Global Empirical Modal Decomposition (EEMD-ANN), and ANN with Discrete wavelet Transform (DWT) decomposition (DWT-ANN), the latter three forecasting models were proposed in their work. The total test data has 364 weekly ED visits data, and the former 80% (in total 291 data) is set as training set and the latter 20% (in total 73 data) is used for testing set according to literature work [16]. The performance metrics of root mean square error (RMSE), mean absolute error (MAE) and correlation coefficient (R) are used to evaluate the forecasting models.

The detail process can be referred to section 3.1 and 3.3.

(1) The time series stationarizing is verified by ADF test and the p-probability value is obtained as 0.5901, which is larger than 0.05 and indicates the non-stationary of the initial time series. After one order of differencing, p-probability value is 0.001 < 0.05 and the time series after differencing can be regarded as stationary, hence d = 1. And the *p* value in white noise test is 1.7261e-05, which indicates the non-while noise series.

(2) ARIMA model identification is to determine the order of AR(p) and MA(q) according to BIC by eq. (3). The calculated BIC matrix is as following.

9 $$ \left[\begin{array}{ccccccc}3.6161& 3.5765& 3.5775& 3.5811& 3.5866& 3.5922& 3.5977\\ {}3.5897& 3.5760& 3.5812& 3.5866& 3.5922& 3.5967& 3.6033\\ {}3.5880& 3.5810& 3.5865& 3.5913& 3.5966& 3.6006& 3.5990\\ {}3.5882& 3.5865& 3.5912& 3.5946& 3.6022& 3.6078& 3.6049\\ {}3.5909& 3.5922& 3.5965& 3.6022& 3.5990& 3.6002& 3.6075\\ {}3.5939& 3.5977& 3.6033& 3.5981& 3.6038& 3.6074& 3.6089\\ {}3.5987& 3.6034& 3.6072& 3.6044& 3.6111& 3.6125& 3.6180\end{array}\right]\times {e}^3 $$

The minimal BIC is 3575 with *p* = 1 and q = 1, and the ARIMA (1, 1, 1) is identified.

(3) Parameters estimation is done by using least-square estimates of coefficients and the estimated parameters are shown in Table 2.

**Table 2:** Parameter estimation and testing of literature case

Parameter	Value	Error	t-Statistic
Constant	2.44949	1.84992	1.32411
AR{1}	0.303464	0.110981	2.73438
MA{1}	−0.722583	0.0795293	−9.08575
Variance	11,995.1	779.512	15.3879

**Fig. 3:**
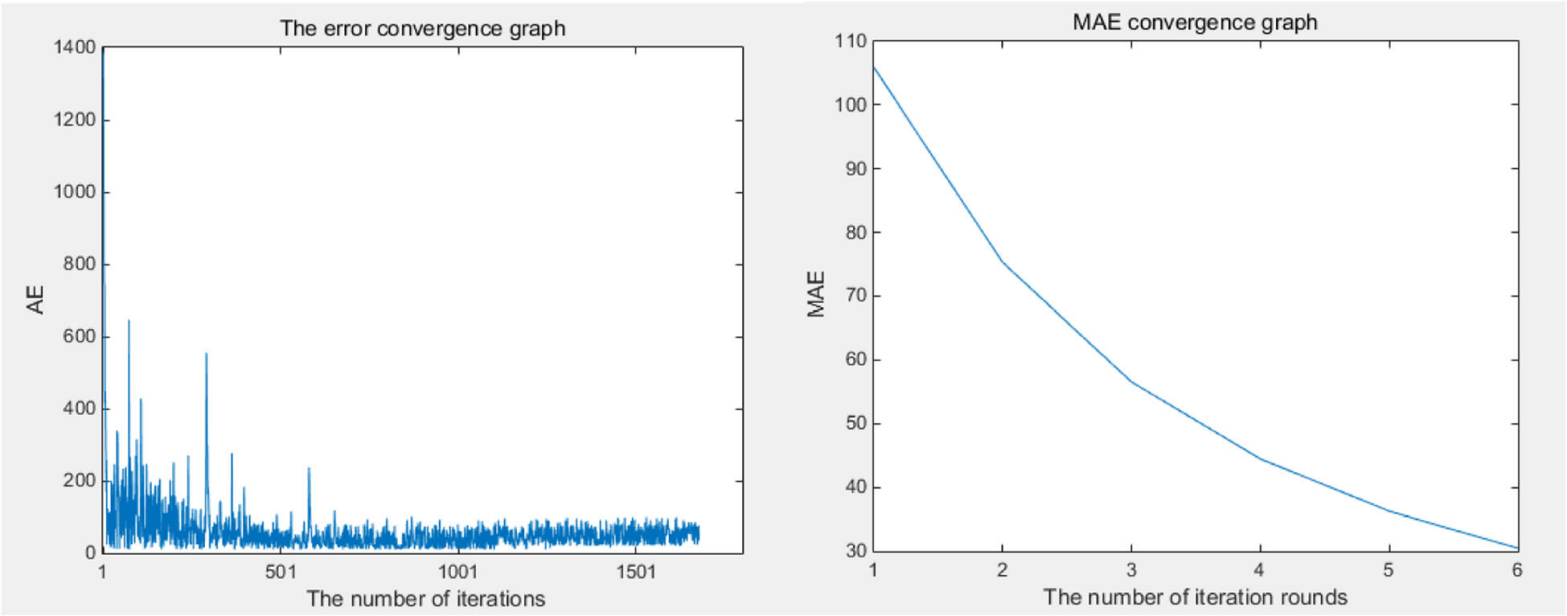
Error convergence in term of iterations and iteration rounds

(4) The estimated parameters are set as the initial weights in self-adaptive filter method, and are further adjusted iteratively according to eq. (6), where the learning rate k is set as $k\le \frac{1}{\underset{n}{\max}\left\{\sum \limits_{i=1}^n{x}_i^2\right\}}$ =9.147582522628832e-08. The self-adaptive adjustment stops until the error converged shown in Fig. 3. It takes additional 1678 iterations (6 rounds of iterations) to obtain the optimal parameters shown in Table 3.

**Table 3:** Comparison before and after adjustment of model parameters in literature test case

Parameter	Before	After
AR{1}	0.303464	0.999314184310874
MA{1}	−0.722583	0.989744187839265

**Fig. 4:**
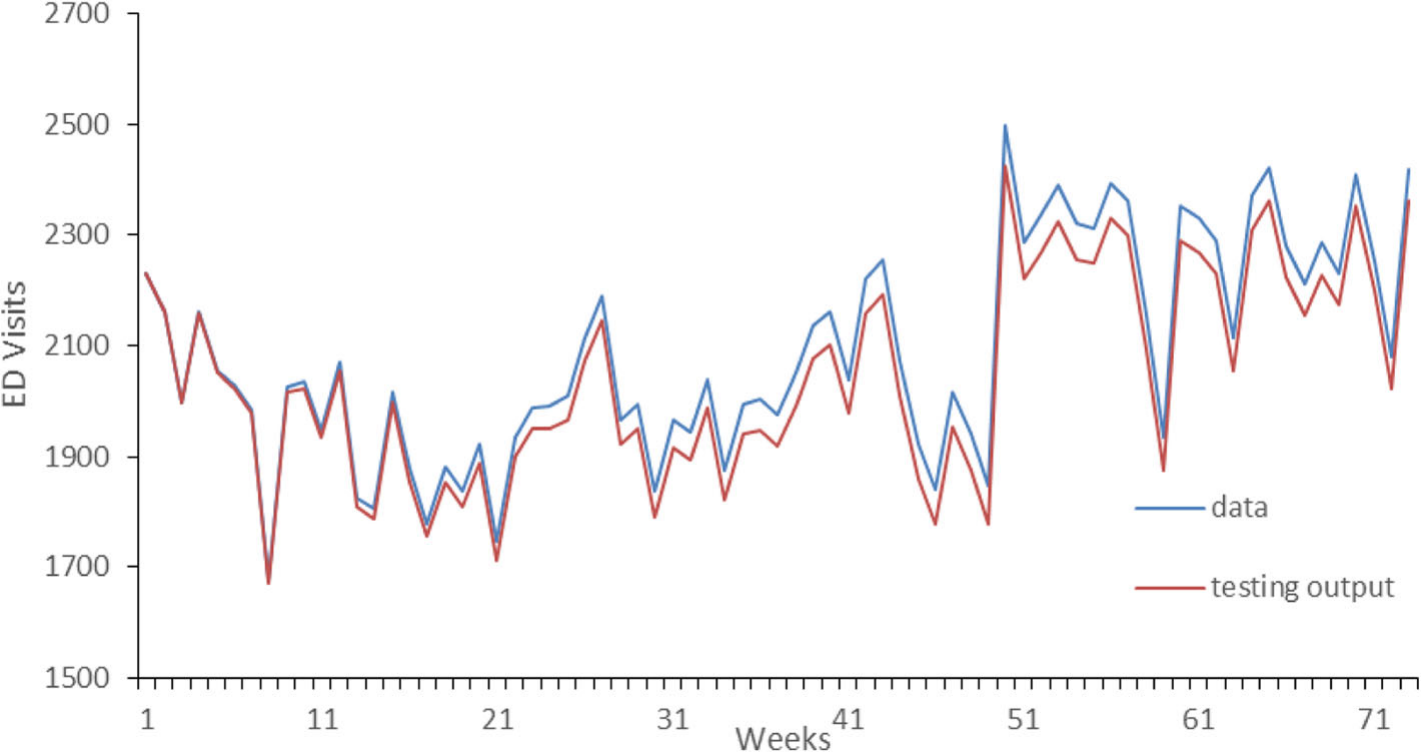
The forecasting results using the hybrid forecasting method

(5) Finally, the final model is used for prediction. The forecasting results are shown in Fig. 4.

The comparison results of hybrid model with other four forecasting methods in Khaldi et al. work [16] are shown in Table 4. As indicated in Table 4, compared with the literature, the proposed hybrid model in MAE outperforms traditional ARIMA 257%, ANN 133%, DWT-ANN 4% and achieves approximately similar accuracy as EEMD-ANN.

**Table 4:** Comparison that results of hybrid model and ANN model

Measurement	EEMD-ANN	DWT-ANN	ANN	ARIMA	Our hybrid model
RMSE	52.86	59.32	149.23	201.73	49.48
MAE	39.88	46.75	104.87	160.70	45
R	0.96	0.95	0.67	0.62	0.98549

### The practical application in medical service demand forecasting

#### Preliminary analysis and pre-processing of data

**Fig. 5:**
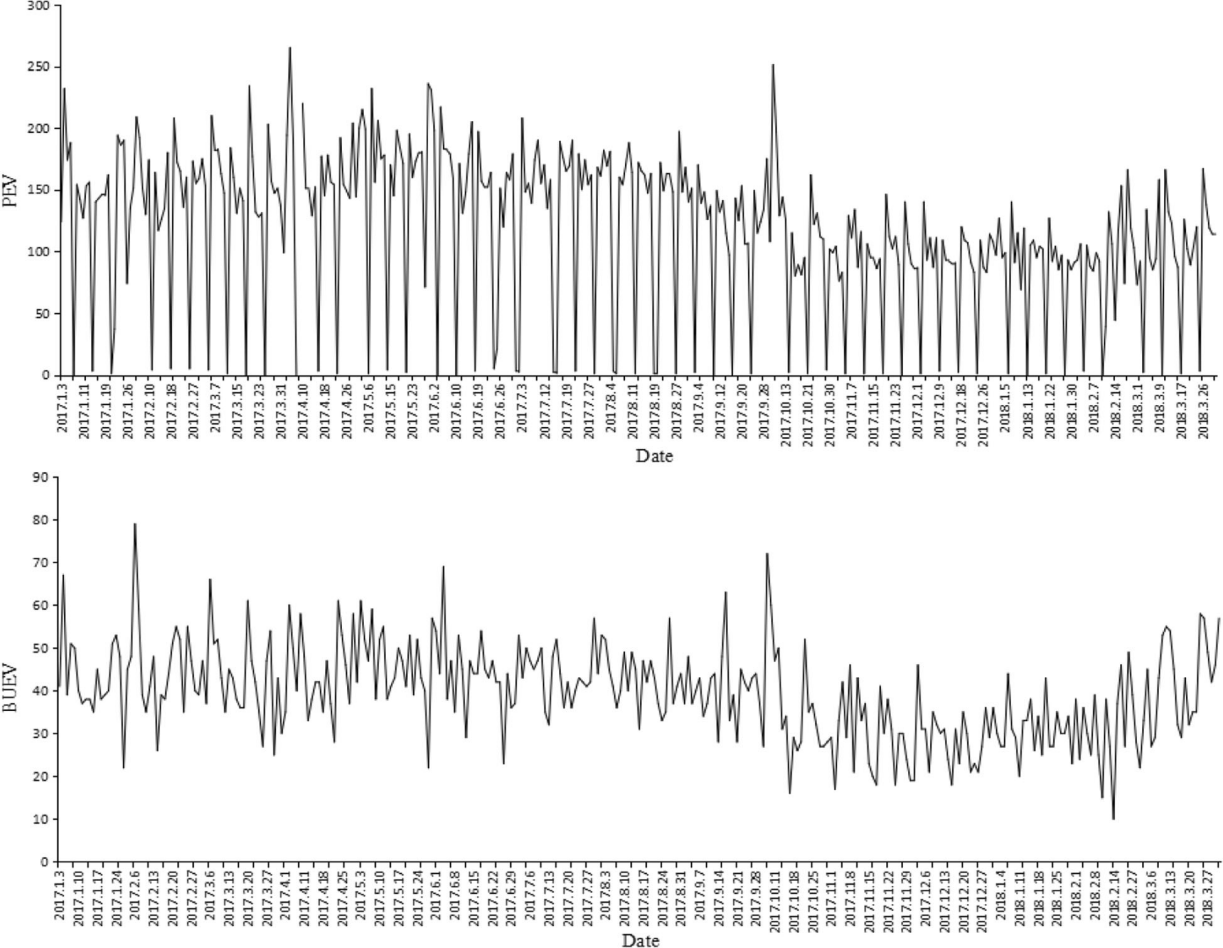
Daily time-series data of PEV and BUEA in January 2017–March 2018. PEV: prenatal examination visits, BUEV: B-ultrasound examination visitors

Daily visits data of the prenatal examination department and the B-ultrasound examination department of a Maternity and Child Health Care Hospital (MCHCH) in Ningbo from January 1, 2017 to March 30, 2018 were collected as the forecasting case data. From January 1, 2017 to March 30, 2018, the total number of prenatal examination visits (PEV) in MCCH was 369. Due to the lack of B-ultrasound examination at weekends and holidays, the total number of the B-ultrasound examination visitors (BUEV) was 310. The time series figure is shown in Fig. 5. We consider the particulars of the weekend and remove the weekend data from the raw data. After the pre-processing, the total number of PEV data and BUEV data is 309.

According to Figs. 5, PEV and BUEV fluctuate greatly, especially on weekends, National Days, Spring Festivals and other holidays. Due to long public holidays during the Spring Festival and National Day, the number of visits was recorded the lowest in February and October. In addition, both two time series data show periodic changes within one week, and there are great differences between different time points in the same cycle. From the time series of data, both PEV and BUEV have no obvious trend. These two time series data belong to time series data containing only periodic features. We removed the data for the first week, and the data was used to build the model, with 283 observations (from January 9, 2017 to on February 28, 2018, *N* = 283, 93% of all data) was the training set. The remaining 22 observations were used to verify the predictive value of the model (T = 22, 7% of all data, from March 1, 2018 to March 30, 2018).

#### Forecasting by ARIMA model and hybrid model

Both forecasting models are implemented in the MATLAB2014a environment.

##### Model fitting and parameter estimation

The time series of PEV and BUEV are stationary sequences and display strong cyclicity feature over the period of a week.

The PEV and BUEV time series data are first undergo the ADF test. From Table 5 the t-statistics and p-probability values of the PEV time series in the ADF test are − 2.2588 and 0.023482, respectively. While the t-statistic value and the *p* value of BUEV are − 2.6585 and 0.0082611. The t-statistics value less than 1% and *p*-value less than 0.05 means the time series stationary. Therefore, both the PEV series and BUEV series are stationary time series and are not necessary for differential operation. Secondly, the white noise tests for PEV and BUEV were performed as well. The results in Table 6 indicate the p-value of the two time series outputs is 0, which means both time series are stationary non-white noise series. Both series did not require to be difference, hence d = 0.

**Table 5:** Unit root test of time series

Department	stat	C Value	P Value
PEV	− 2.2588	−1.9418	0.023482
BUEV	−2.6585	−1.9418	0.0082611

**Table 6:** White noise test of time series

Department	stat	C Value	P value
PEV	419.93	12.5916	0
BUEV	169.9278	12.5916	0

The p and q order of the ARIMA model is determined according to the BIC value calculated by eq. (3).

All combinations of ARIMA (p, q) when p and q are both less than log (length (data)) = 6 (where length (data) is the length of the total data amount of the time series) are calculated and the BIC values are shown in BIC matrix, where row represents p value from 0 to 6, while column represents q value from 0 to 6. The PEV BIC matrix and the BUEV BIC matrix are shown in (10, 11). As indicated in these two matrix, the minimum PEV BIC value is 2804.5322 with *p* = 1 and q = 1, and the minimum BUEV BIC value is 2109.1708 with p = 1 and q = 1. Hence, the fitted models of PEV and BUEV are both ARIMA (1, 0, 1) and the estimated parameters using least-square estimates of coefficients for the two models are shown in Table 7. The ARIMA models of PEV and BUEV have *p* values greater than 0.05 according to the residual analysis, which indicate the reliability of both models.

10 $$ \left[\begin{array}{ccccccc}2.961& 2.9094& 2.8831& 2.8760& 2.8764& 2.8673& 2.8665\\ {}2.8683& 2.8045& 2.8083& 2.8130& 2.8184& 2.8229& 2.8286\\ {}2.8427& 2.8102& 2.8077& 2.8062& 2.8107& 2.8161& 2.8212\\ {}2.8392& 2.8138& 2.8054& 2.8140& 2.8188& 2.8194& 2.8194\\ {}2.8379& 2.8186& 2.8107& 2.8184& 2.8191& Inf& 2.8283\\ {}2.83292& 2.8238& 2.8158& 2.8240& 2.8286& Inf& Inf\\ {}2.8369& 2.8293& 2.8205& 2.8572& 2.8140& Inf& 2.8656\end{array}\right]\times {10}^3 $$

11 $$ \left[\begin{array}{ccccccc}2.1925& 2.1720& 2.1665& 2.1617& 2.1670& 2.1641& 2.1639\\ {}2.1607& 2.1092& 2.1144& 2.1200& 2.1257& 2.1262& 2.1316\\ {}2.1513& 2.1148& 2.1200& 2.1256& 2.1310& 2.1309& 2.1684\\ {}2.1468& 2.1203& 2.1257& 2.1258& 2.1307& 2.1467& 2.1523\\ {}2.1508& 2.1554& 2.1313& 2.1299& 2.1361& 2.1265& 2.1350\\ {}2.1465& 2.1280& 2.1318& 2.1347& 2.1294& 2.1253& 2.1252\\ {}2.1485& 2.1336& 2.1399& 2.1590& 2.1351& 2.1252& 2.1426\end{array}\right]\times {10}^3 $$

**Table 7:** Parameter estimation and testing of PEV and BUEV

	Parameter	Value	Error	t-Statistic
PEV	AR{1}	0.995535	0.00972474	102.371
	MA{1}	−0.883045	0.0328523	−26.8792
BUEV	AR{1}	0.997307	0.00903517	110.381
	MA{1}	−0.931795	0.028666	−32.5053

*PEV* prenatal examination visits, *BUEV* B-ultrasound examination visitors

##### Model parameter adjustment

The formula (6) in the adaptive filtering method is applied to adjust the parameters. The error converges after 576 iterations in PEV and 286 iterations in BUEV. Table 8 shows the comparison before and after the adjustment of the model parameters.

**Table 8:** Comparison before and after adjustment of model parameters in the PEV and BUEV

Parameter	PEV		BUEV	
	Before	After	Before	After
AR{1}	0.995535	1.000919	0.997307	1.012281
MA{1}	−0.883045	− 0.997426	−0.931795	− 0.99602

*PEV* prenatal examination visits, *BUEV* B-ultrasound examination visitors

##### Model forecasting

**Fig. 6:**
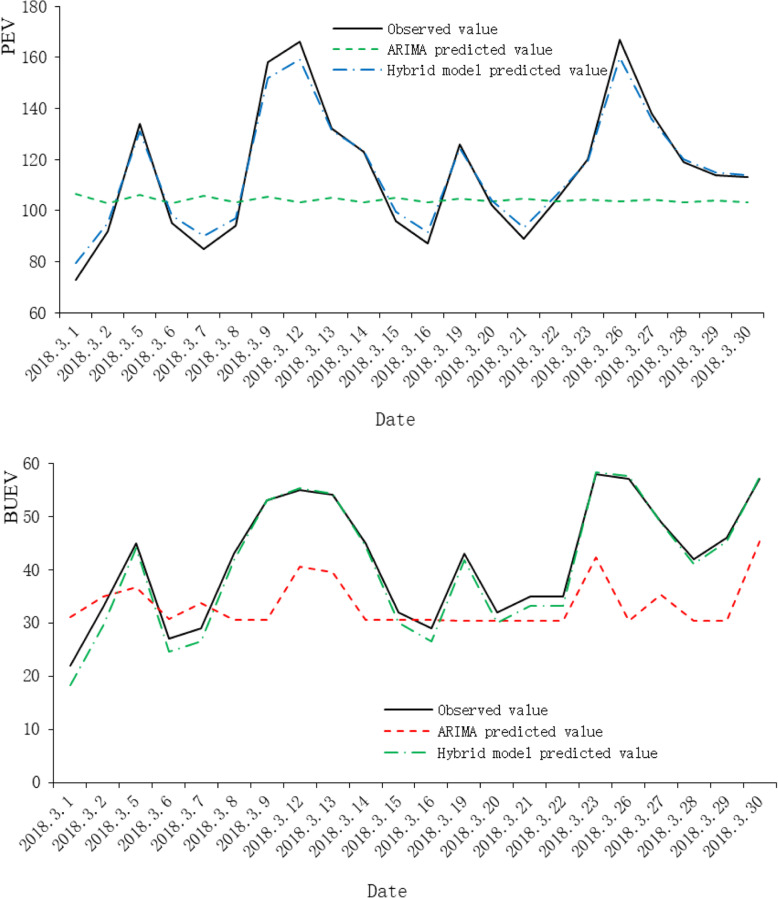
Comparison of PEV and BUEA forecasting values and observed values. ARIMA: auto-regressive integrated moving average, Hybrid model: ARIMA-self-adaptive filtering hybrid forecasting model, PEV: prenatal examination visits, BUEV: B-ultrasound examination visit

Finally we applied the fitted two models to predict the PEV and BUEV time series and obtained a 22-day predicted value (Figs. 6).

As shown in Table 9, the predicted values of the ARIMA model and the hybrid prediction model are compared. Similarly, we compare the PE_max_, PE_min_, MAPE and *σ*_*PE*_ values of the predicted results. In order to more intuitively represent the difference between the predicted value and the actual observed value, Fig. 6 respectively show the fitting effect of the predicted values of PEV and BUEV.

**Table 9:** Comparison of the forecasting performance of the ARIMA and Hybrid model

	ARIMA		Hybrid models	
	PEV	BUEV	PEV	BUEV
PEmax(%)	41.7%	47.64%	6.16%	2.14%
PEmin(%)	2.39%	4.70%	0.04%	0.71%
MAPE(%)	18.53%	27.69%	2.79%	1.25%
σ_PE_(%)	11.74%	15.39%	1.87%	0.38%

*ARIMA* auto-regressive integrated moving average, *Hybrid model* ARIMA-self-adaptive filtering hybrid forecasting model, *PE* percentage error, *PEV* prenatal examination visits, *BUEV* B-ultrasound examination visitors, *PE*_*max*_ Maximum percentage error, *PE*_*min*_ Minimum percentage error, *MAPE* mean absolute percentage error, *σ*_*PE*_ value of the standard deviation of the PE

As mentioned above, in the two case studies, the analysis results show that the hybrid model has better prediction performance, and the mean value of the variance of the relative error and relative error is smaller.

## Discussion

An efficient forecasting model has to have a tradeoff between prediction accuracy and model complexity (number of parameters). In this paper, we choose the basic ARIMA model to identify the features of the time series, describe the autocorrelation, trend and periodicity from the time series, and establish estimate parameters. Then the parameters of the model are further adjusted by the steepest descent principle in the adaptive filtering method to keep tracing and immediately feedback to compensate error. Although it takes additional time to optimize ARIMA parameters, the forecasting accuracy is greatly increased because of such self-adaptive adjustment iteration. The cost of the additional computation is case by case.

As indicated in section 4.1 and 4.2, for different test cases in TDSL benchmark library, the literature test case, and the practical application in outpatient visits in MCHCH in Ningbo China, the proposed hybrid forecasting method outperforms several other methods. The results show that the hybrid model has shown obvious improvement in forecasting accuracy compared with the traditional ARIMA model case by case. Furthermore, when dealing with the time series display strong cyclicity feature over the period, our hybrid method even reports better performance. The traditional ARIMA model predicts 18.53% MAPE value of PEV, while the hybrid model is 2.79%. For the BBUEV time series, the MAPE value of the ARIMA model is 27.69%, and the hybrid model is 1.25%. It can be seen from Figs. 6 that the prediction results of the ARIMA model change relatively smoothly, and the trend of data changes is not well shown. Referring to Fig. 2, the predicted results of the two models are compared with the observed values. The results of the hybrid prediction model proposed in this paper are more detailed than the ARIMA model and are closer to the actual situation.

What’s more, the PEV and BUEV of MCHC in Ningbo are further studied using the proposed hybrid forecasting model. According to the characteristics of the time series, both PEV and BUEV are in a cycle of one week, due to the large number of patients and limited medical resources. The medical service demand of most health care hospital in China has a weekly cycle, so this model can be extended to other hospitals. And data size is extended to medium and long-term data, this model can also be applied to medium or long time forecast.

## Conclusions

In this work, an integration of a traditional ARIMA model and a self-adaptive filtering model is proposed to forecast the demand of medical service. ARIMA is used to identify the demand feature and obtain the initial prediction model, while self-adaptive filtering model is applied to re-adjust the prediction model weights to further improve prediction accuracy. Such hybrid prediction model has advantage in both prediction accuracy and prediction horizon. Applied to the forecasting on daily outpatient visiting of Maternal and Child Health Hospital in Ningbo, the hybrid model outperforms to the traditional ARIMA model in prediction accuracy. The MAPE predicted by the traditional ARIMA model in the two departments of prenatal examination and B-ultrasound examination is 18.53 and 27.69%, and that of the hybrid model is 2.79 and 1.25%, respectively. The results of this forecasting study can be later used in outpatient appointment scheduling decisions of the target Maternal and Child Health Hospital to optimize the pregnant appointment, alleviate long queues in outpatient clinics and increase patient satisfaction.

This article studies the forecasting of medical service demand. With an accurate demand forecasting, the resource can be appropriately allocated and assigned to match the forecasting demand so as to in one hand reduce the patients waiting time and in the other hand reduce the medical staffs’ idle time. Furthermore, the accurate forecasting of daily outpatient visits is also crucial to scientific management of the medical service provider since it is actually the critical input to several decisions in system operation, e.g. material resource planning and scheduling, inventory control, resource allocating, labor resource rostering, etc.

In addition, the proposed hybrid model enhances the capability of traditional ARIMA, while keeps the advantage of its simplicity in only utilizing endogenous time series data as input. It can also be applied to other application fields as long as there has the time series forecasting problem.

There are still some limitations and several further studies need to be done in this research. Firstly, for the practical application of the hybrid model, only the outpatient data of two specific departments in one Ningbo Maternal and Child Health Hospital for the next 4 weeks is predicted, which is still a kind of a short-term forecasting. The mid-term and long-term medical service demand forecasting needs to be further verified using hybrid model. The follow-up study needs to deeply analyze the best application conditions of the hybrid model. Secondly, as revealed in several researches [16, 36], data pre-processing is needed because data in the real world is incomplete, noisy and inconsistent, the couple with pre-processing technique onto data can improve forecasting capabilities of the ARIMA model. Hence, instead of directly using the inherent features of single visiting number time series data as input, the data preprocessing method like decomposing the time series and other data series like climate factors may also be involved in the forecasting model to further improve the predicting accuracy.

## Data Availability

All data generated or analysed during this study are included in this published article [and its supplementary information files].

## Abbreviations

ARIMA: Autoregressive Integrated Moving Average
TSDL: Time Series Data Library
MCHCC: Maternal and child health care center
MAPE: Mean Absolute Percentage Error
ED: Emergency Department
MA: Moving Average
ES: Exponential Smoothing
ANN: Artificial Neural Networks
SVM: Support Vector Machines
IPD: Indoor Patient Department
OPD: Outdoor Patient Department
LOS: Length of Stay
FWNN: Feedforward Neural Network
RNN: Recurrent Neural Network
ACF: Auto-correlation function
PACF: Partial auto-correlation function
MAE: Average absolute error
PE: Prediction Errors
PEV: Prenatal Examination Visits
BUEV: B-ultrasound examination visitors
AR: Autoregressive
